# Discussing patient preferences for levels of life-sustaining treatment: development and pilot testing of a Danish POLST form

**DOI:** 10.1186/s12904-021-00892-2

**Published:** 2022-01-11

**Authors:** Lone Doris Tuesen, Hans-Henrik Bülow, Anne Sophie Ågård, Sverre Mainz Strøm, Erik Fromme, Hanne Irene Jensen

**Affiliations:** 1 Department of Anaesthesiology and Intensive Care, Vejle and Middelfart Hospitals, University Hospital of Southern Denmark, Beriderbakken 4, DK-7100 Vejle, Denmark; 2 Department of Regional Health Research, University of Southern Denmark, J.B.Winsløwsvej 19, DK-5000 Odense, Denmark; 3 Department of Anaesthesiology and Intensive Care, University Hospital Holbaek, Holbaek, Denmark; 4 Department of Intensive Care, Aarhus University Hospital, Palle Juul-Jensens Boulevard 100, DK-8200 Aarhus N, Denmark; 5 Department of Public Health, Aarhus University, Bartholins Allé 2, DK-8000 Aarhus C, Denmark; 6 Medical Center, Aeblehaven 1, DK-5580 Norre Aaby, Denmark; 7 Dana-Farber Cancer Institute, 450 Brookline Ave, Boston, MA 02215 USA

**Keywords:** Advance care planning, End-of-life, Shared decision-making

## Abstract

**Background:**

Medically frail and/or chronically ill patients are often admitted to Danish hospitals without documentation of patient preferences. This may lead to inappropriate care. Modelled on the American Physician Orders for Life-Sustaining Treatment (POLST) form, the purpose of the study was to develop and pilot test a Danish POLST form to ensure that patients’ preferences for levels of life-sustaining treatment are known and documented.

**Methods:**

The study was a mixed methods study. In the initial phase, a Danish POLST form was developed on the basis of literature and recommendations from the National POLST organisation in the US. A pilot test of the Danish POLST form was conducted in hospital wards, general practitioners’ clinics, and nursing homes. Patients were eligible for inclusion if death was assessed as likely within 12 months. The patient and his/her physician engaged in a conversation where patient values, beliefs, goals for care, diagnosis, prognosis, and treatment alternatives were discussed. The POLST form was completed based on the patient’s values and preferences. Family members and/or nursing staff could participate. Participants’ assessments of the POLST form were evaluated using questionnaires, and in-depth interviews were conducted to explore experiences with the POLST form and the conversation.

**Results:**

In total, 25 patients participated, 45 questionnaires were completed and 14 interviews were conducted. Most participants found the POLST form readable and understandable, and 93% found the POLST form usable to a high or very high degree for discussing preferences regarding life-sustaining treatment. Three themes emerged from the interviews: (a) an understandable document is essential for the conversation, (b) handling and discussing wishes, and (c) significance for the future.

**Conclusion:**

The Danish version of the POLST form is assessed by patients, families, physicians, and nurses as a useful model for obtaining and documenting Danish patients’ preferences for life-sustaining treatment. However, this needs to be confirmed in a larger-scale study.

**Supplementary Information:**

The online version contains supplementary material available at 10.1186/s12904-021-00892-2.

## Background

As populations in western countries live longer, the rate of chronic serious illnesses is increasing [1]. Along with medical progress, this has caused an increase in the admission of patients with limited life expectancies to intensive care units (ICUs) or acute care facilities [2–5], and physicians are often confronted with decisions to withhold or withdraw treatment [6]. Studies examining end-of-life (EOL) practices in ICUs have shown that patients’ wishes are often not known [7, 8]. A Canadian study surveyed medical patients primarily above 80 years of age, and only 30% of their expressed wishes for the level of treatment were documented in concordance with the medical records [9]. The COVID-19 pandemic has added an increased need for knowing and honouring patients’ wishes, as those with advanced illness and frailty are the population most likely to develop severe symptoms and have a higher death rate [10, 11]. Not knowing patients’ wishes may lead to inappropriate care [12, 13].

A study from 2018, examining patient preferences regarding shared decision making in the emergency department, concluded that 98% wanted to be involved in decisions when “something serious is going on” [14]. However, many patients feel unable rather than unwilling to engage in decision-making. Their wish not to cause any inconvenience and be a “good” patient could override their desire for shared decisions. This could be mistaken for a lack of interest in engaging in decision-making [9, 14, 15].

Different models to clarify patients’ wishes and preferences exist such as the British ReSPECT [16], the American Physician Orders for Life Sustaining Treatment (POLST) [17], and a variety of other advanced care planning tools [18]. One of the most widely used and researched advanced care planning tools is the POLST, which is a primary tool in honouring patients’ treatment preferences in large parts of the American healthcare system. The POLST form covers the services offered by general practitioners, hospitals, and nursing homes. The POLST form is completed based on a process of shared decision-making. During the conversation between the patient, the physician, and perhaps family members and nursing staff, the patient shares his or her values, beliefs, and goals for care, and the healthcare professional explains the patient’s diagnosis, prognosis, and treatment alternatives, including the benefits and limitations of life-sustaining treatment. Together they reach an informed decision about the desired treatment, based on the patient’s values, beliefs and goals for care, and their completed POLST form highlights the treatment preferences identified through the conversation [19–22]. Patients in Oregon with POLST Comfort Measures Only orders were much less likely to die in hospitals than patients without POLST forms or with POLST orders for Full Treatment [20].

Patient involvement, including shared decision-making, has become a key topic in healthcare in the Western World [23, 24]. Even so, presently the Danish healthcare system has no standard procedure to formalize healthcare professionals’ conversations with patients about treatment options, values, and preferences. Despite the growing political wish to support patient choice, incorporating a more patient-centered approach to life-sustaining treatment is still a challenge for healthcare professionals [25].

The purpose of the study was to develop and pilot test a Danish POLST form to ensure that patients’ preferences for levels of life-sustaining treatment are known and documented.

## Methods

The study was a mixed methods study, designed as an explanatory sequential study with combined quantitative and qualitative data [26] and consisting of two steps: 1) the development of a Danish POLST form and 2) a pilot test of the Danish POLST form in different patient settings, evaluated by questionnaire and in-depth interviews. The study sites were home care, nursing homes, hospitals, and general practitioners’ clinics, and the term “patient” covers participants from all four types of sites.

### Developing a Danish POLST form

The Danish POLST form was developed on the basis of literature and recommendations from American POLST sources [16, 27, 28]. To achieve more in-depth knowledge about the American POLST, a study visit to America was conducted to meet POLST key persons in Oregon, California, and West Virginia.

The first draft of the Danish POLST was assessed and adjusted from May to October 2017 by four patients, four family members, three physicians, four nurses, and the project advisory board. The advisory board consisted of 14 national and international healthcare professionals with expert knowledge regarding EOL issues and patient involvement together with a patient and a family member representative. The title of the Danish form is “Patient-and-Physician Decisions for End-of-Life” which in Danish also provides the acronym POLST (the form is available in a non-validated translation as additional file A1). The American POLST varies from state to state; for example, some states include a section for antibiotics and not all states include artificial nutrition. In September 2019, a national POLST form was released in the US, which reduced the variation among forms. Like some of the American POLST forms, the Danish POLST form includes sections regarding three areas: cardiopulmonary resuscitation (CPR), level of treatment (full, selective, or comfort measures only), and artificial nutrition. As shown in Table 1, there are some differences between the American and Danish POLST forms.

**Table 1:** Differences between the American and Danish POLST forms

POLST USA	POLST Denmark
The POLST form is always voluntary and is usually for persons with serious illness or frailty.	The POLST form is always voluntary and is for persons with serious illness or frailty.
For persons at any age with serious illness and/ or limited life expectancy and/or a person who has a disease process (not just a disability) that is an end-stage medical condition or terminal illness.	For persons of 18 years and older with no known cognitive impairment, with serious illness or frailty for whom their physician would not be surprised if they died within a year.
Can be completed by the patient or their surrogate with a licensed health professional	Can be completed by the patient and a physician (MD)
Actionable medical orders for current treatment	This document is currently in testing and, therefore, cannot serve as an actionable medical order (decisions are additionally registered in the patient’s medical record and are thereby actionable medical orders).

### Participants and settings

To include a diverse range of participants, a heterogeneous purposive sampling was used [29] with study sites in hospital wards, general practitioners’ clinics, home care, and nursing homes from four out of five Danish regions. The first author visited all sites, introduced the project to all relevant staff members, and supplied written instructions and project material. After the introduction, staff members at the sites identified suitable patients. Participants eligible for inclusion were patients with serious illness and/or frailty for whom the physician would not be surprised if the patient died within the next 12 months. The participants were 18 years and above with no known cognitive impairments. As this was a pilot study, 25 participants were assessed as an appropriate sample size. According to Danish law, surrogates cannot make treatment decisions on behalf of incapacitated patients.

### Conversation

The physician facilitated a conversation with the patient based on the POLST form. Depending on the patients’ wishes, one or more family members and/or nursing staff could participate. The nurses were mainly passive participants but if needed helped to facilitate the dialogue and afterwards follow up on the conversations. The conversation included the patient’s values, beliefs, goals for care, as well as their diagnosis, prognosis, and treatment alternatives. The POLST form was completed based on the patient’s values and preferences. As the POLST form is still a project document in testing and, therefore, not a legal document, the wishes were also documented in the patient’s medical records. The healthcare professionals did not receive specific education in conducting a POLST conversation, but the project material included a list of “helpful prompts and questions” to initiate, conduct, and conclude the conversation.

### Evaluation

Questionnaires and in-depth interviews with a purposive selection of participants were used to identify perspectives on the POLST form itself, experiences with the conversation, and actual treatment preferences. Patients and participating family members received a short questionnaire 7 days after the conversation, whereas physicians and nurses received the questionnaire when their last patient conversation had concluded to prevent multiple responses. The study was conducted between November 2017 and July 2018.

#### Questionnaire

The questionnaire used in the evaluation was initially based on POLST survey instruments [30] and a Danish version of the Consult Decision Aid Prototype [31]. The questions were then assessed and adjusted in collaboration with patients, family members, physicians, nurses, teachers in questionnaire methodology, and the advisory board to test for face and content validity [32]. The questionnaire consisted of nine questions and was similar for all participant groups, except for questions about background characteristics. The first types of questions were yes/no questions, for example: “Do you find the POLST document understandable? The second type of questions had responses on a five-point Likert scale (to a very high degree, to a high degree, to some degree, to a low degree, not at all and not relevant) and, for all questions, comments with ideas and recommendations could be added. The questionnaire is available in a non-validated translation as additional file A2. All patients and family members could fill in the questionnaire on paper and return it in a prepaid and addressed envelope, or receive and complete the questionnaire via email through the online system REDCap (Research Electronic Data Capture). All physicians and nurses received the questionnaire by email/REDCap at their place of employment. If questionnaires had not been returned within 3 weeks, a reminder was sent by email or placed by phone.

#### Interviews

The semi-structured interview guide was based on methodological literature [33] as well as on the assessments and comments from the questionnaire and was pilot tested on all groups of participants at an outpatient clinic. The structuring of the interview guide was inspired by Kvale’s interview criteria [33] and focused on thoughts on and perceptions of the POLST form, experiences with the POLST conversation, and preferences regarding wishes for levels of treatment. The interviews were conducted based on a heterogeneous purposive sampling, to include different settings and age groups [29]. The individual participant’s questionnaire responses were then used to nuance the interviews. Interviews were conducted at one hospital ward, one home care setting, and one general practitioner’s clinic, as well as two nursing homes in rural areas and one in a city. Two interviews were conducted as telephone interviews. All interviews were audio-recorded and transcribed.

### Data analysis

Quantitative data were analysed using descriptive statistics. Interview data were analysed by the first and last authors using systematic text condensation as described by Malterud [34, 35]. The four steps of analysis comprised: (I) reading all the interview material several times to gain an overall impression, (II) identifying meaning units representing different aspects of participants’ experiences with the POLST form, and the conversation and coding for these, (III) condensing the contents of each of the codes groups, and (IV) synthesizing the contents of each code group to generalize descriptions and themes concerning the POLST form and the conversation [35]. The computer software QSR NVivo Pro 12 was used for organizing the coding and analysis of the qualitative data.

## Results

A total of 25 out of 28 patients invited to engage in a POLST conversation accepted the invitation. Additionally, nine family members, seven physicians, and six nurses participated in the conversations. The mean patient age was 82 (range 58–96) and 76% were female. A total of 21 (84%) patients chose no CPR, 8 (32%) chose comfort measures only, 16 (64%) chose selected treatments, 1 (4%) chose full treatment and 20 (80%) did not want artificial nutrition.

### Questionnaire

The response rate was 92% for patients (23/25), and 100% for physicians, family members, and nurses. A total of 90% of the patients and 100% of physicians, family members, and nurses found that the POLST form was readable and understandable, 82% of the patients found that the level of information was appropriate and 87% of the patients and 100% of the other participants did not find that there was information that should either be added or removed. As shown in Table 2, the majority of all participants found, to either a high or very high degree, that the POLST document was usable for conversations about wishes for life-sustaining treatment.

**Table 2:** Was the POLST form usable to discuss wishes for levels of life-sustaining treatment?

	All	Patients	Physicians	Family	Nurses
			n (%)		
To a very high degree	20 (44)	8 (35)	2 (29)	5 (56)	5 (83)
To a high degree	21 (47)	12 (52)	4 (57)	4 (44)	1 (17)
To some degree	2 (5)	1 (4)	1 (14)		
To a lesser degree					
Not at all					
Not applicable	2 (4)	2 (9)			

Comments elucidated the quantitative responses: “*It provides an opportunity to think and to verbalize while I am still able to do so”* (patient), “*I am happy that my children now also know my wishes”* (patient), and *“It is a clear document in plain language”* (physician).

A physician commented that all the project tasks, such as providing information, obtaining informed consent and project registration, were time-consuming, not so much the POLST conversation in itself.

A number of the participating settings did not have guidelines for how and where patients’ wishes should be documented, and participation in the study entailed improved documentation practice, in fact now acting according to Danish national health law.

### Interviews

Interviews were conducted with four patients, three physicians, three family members, and four nurses, representing different settings and age groups.

Three main themes were identified within all four groups:

A. An understandable document is essential for the conversation.

The majority of the participants pointed out the importance of a manageable and clear document. A patient said: *“It was set up in a way that was easy to understand. It wasn’t with all sorts of different questions and too much text. It was set up fine”.*

One family member would have liked to have been better prepared for the content of the conversation, but all family members experienced a satisfying explanation of the POLST form by the physician.

The two staff groups found the POLST form to be readable and understandable. A physician emphasised: *“…. Here it is very clear what you have to do. I think there is a difference in the way I ask the questions and also spend time because it is so systematic and because there is this clarity that this is what we are talking about”.*

A nurse added: *“…. clear even for nursing home residents who may not find it easy to acquire new expressions, or have difficulty understanding our professional language at all”.*

B. The potential of explicating wishes for levels of treatment

All patients expressed relief in having voiced and documented their wishes. A patient stated: *“Now I feel at peace, it’s as if a stone fell from my heart”.*

Family members overall expressed that the conversation provided insight into the patients’ wishes for levels of life-sustaining treatment. For some, being confronted with the risk of their loved one dying was an emotional experience. At the same time, all family members expressed their support of the patients and their wishes. A patient’s son said: *“It is good to know my mother has made her decisions based on a conversation with her physician”.*

The physicians agreed that most patients welcome a conversation about levels of life-sustaining treatment. A physician said: *“The patients know that this is something we need to talk about”.*

However, some physicians and nurses found that facilitating a conversation about life-sustaining treatment decisions depended on departmental and national culture. A physician stated: *“This (POLST conversation) should preferably be founded in a culture where this is something you can talk about. And if you can’t, then it (the culture) must be established before it starts…the challenge is how dare we start the conversation? How do we overcome this?”*

III. A conversation about decisions is crucial for the future

One of the challenges regarding levels of treatment was to clarify the benefits and burdens of the different options.

The patients saw the physicians as consultants, giving them information to consolidate their decisions. A nursing home resident said: *“It was a good conversation where the doctor explained what the different choices meant…. I did not know they could do so much to help me”.*

One physician found that the clarification was the challenging part: *“The next question (about the level of treatment) is actually the most difficult: How much treatment do they want…. Whether it is comfort-focused treatment, plus a little extra, or it is full treatment. Some patients do not want to be resuscitated, yet they want full treatment…. This proves to me that it is necessary that this is a dialogue, so I can explain to the patient what the preferences entail”.*

All nurses mentioned that the POLST conversation dealt with patient safety by gaining knowledge of how the patient wished to be helped, as well as by informing the patient as to how they could be helped. Furthermore, the nurses emphasised the importance of having the discussion and decisions documented: *“Today, as the citizen’s condition worsens, you consult the physician on call, who then maybe doesn’t know the citizen…………. so I think it is good for both the citizen himself and the family and the nursing home that this is assured”.*

All family members stressed the assurance that decisions made were in accordance with the patient’s wishes. A patient’s daughter stated: *“My mother is dead…. My siblings and I are so grateful that mom was in the POLST project…that way we could stand together on mother’s last wishes…. we all get along well, but I don’t think we necessarily would have agreed on what should have been done for mother had it not been for the POLST form”.*

## Discussion

Most participants found the POLST form readable and understandable, and commented that it was usable either to a high or very high degree, for discussing patients’ preferences for levels of life-sustaining treatment.

Regarding preferences for levels of treatment, only one patient out of 25 chose full treatment. This emphasises the necessity to clarify treatment needs among patients who are likely to die within 12 months. If this is not clarified, patients may against their wishes receive excessive treatment at the last stage of their life [13, 36]. The POLST form is a medical order, but using a POLST form or other structured tools such as the PREPARE Advanced Directive [37], the ReSPECT [16], or the Respecting Choices® [38] can help initiate the clarification. The POLST form is widely used in the US and prior research suggests that POLST facilitates concordance between medical orders and preferences for life-sustaining treatment [19] and that wishes documented on POLST forms are largely concordant with care delivered [39]. Critiques of the POLST include the risk of it being filled in without a conversation about values and goals, not allowing for changes in preferences, and lack of clarity in interpretation of preferences [40].

The majority of patients in the current study chose no CPR and selective or palliative measures only. This is in line with other studies: A Canadian study where 12% wanted full treatment, 20% abstained from CPR, 30% wanted just comfort and 30% wanted a mix of no CPR and comfort [9], and an Australian randomized study of +/− advanced care planning among older patients. In this study, one of the ACP patients died in the ICU although 75% opted for life-prolonging treatment before the ACP interview [41]. The fact that the majority of patients preferred no CPR and selective or palliative measures only, as opposed to full treatment, is also in accordance with American POLST research [20]. However, a recent study comparing two decedent cohorts of Oregon POLST orders from 2010 to 2011 and 2015–2016, respectively, showed an increase in orders for more aggressive life-sustaining treatment as more patients are completing POLST forms with their healthcare professionals [42].

For many healthcare professionals, initiating a conversation about EOL is considered problematic and one of the reasons given is that it may be “too much” for the patient to handle [43]. This is, however, in contrast to a number of different studies suggesting that a substantial number of patients wish to engage in this conversation [14, 44]. The majority of the participating physicians found the POLST form helpful for both initiating and conducting EOL conversations, as the POLST form made it clear what needed to be discussed. Providing sufficient information and clarifying the possible consequences of different choices can be challenging, and the conversations need to include patient goals and values before decisions about life-sustaining treatments can be made [10].

Lack of time is mentioned as a barrier for conducting EOL conversations [45]. However, an American study showed that the median time for a conversation about goals of care with critical care survivors requiring prolonged mechanical ventilation only was 15 min [46]. In the current study, a physician commented that the conversation itself was not time-consuming. This does not mean that EOL conversations are always easy or quick, but it suggests that the barriers anticipated by healthcare professionals may not be as significant as they perceive.

Ensuring that the patient’s wishes are documented and known by the healthcare professionals taking care of them is essential [9, 47]. In this study, we found that the documenting patients’ wishes for levels of life-sustaining treatment were frequently lacking, despite demand for this in Danish law. That documentation is now practiced due to participation in the study improved patient safety, as failing to honor the patient’s known wishes is to be considered a medical error (malpractice) [48], which may lead to inappropriate care [13].

Strengths of the study include the involvement of patients and family members throughout the study planning and development of the POLST form and instrument, the high response rate, the inclusion of different patient settings, and the mixed methods, which provided nuanced results. The American POLST was used as inspiration for the Danish Form because it is short and precise, it has been used for a number of years in different settings and it has been subjected to substantial research. A number of patients and citizens from different settings and varying social backgrounds were involved in the development and testing of the Danish POLST form to ensure that the content was understandable and made sense in a Danish context. Other advanced care tools may also have been applicable, but the current study suggests that having a form in itself promotes conversations about wishes regarding the level of treatment.

Limitations include the limited number of participants, 76% of the patients being female, and the risk of selection bias as patients were included at the staffs’ discretion who were likely to have been assessed as being well suited to inclusion in the study. Therefore, the participants may not be representative of the total population, and further studies currently being undertaken will focus on including a wider spectrum of patients. However, it is noteworthy that the results from the current study are similar to those from a larger Canadian study [9]. The study only included patients with decision-making capacity. Advanced care planning is equally important for patients without decision-making capacity, but in Denmark, this cannot be achieved based on a POLST form, as surrogates do not have legal rights to make decisions on behalf of the patient. The generalisability of the current study therefore only concerns patients with decision-making capacity. The physicians were instructed to discuss the patients’ values, beliefs and goals before filling in the POLST. However, the conversations were not monitored, and the quality of the conversations may have varied. Furthermore, the participation of nurses was unmonitored, and it is unknown whether their involvement influenced the individual decisions made by patients, or to what extend the nurses were important in achieving high rates of satisfaction with the process. The questionnaire used in the present study was developed for this specific study, as we were unable to find a validated specific questionnaire. To secure content validity, the questionnaire was developed based on both American and Danish validated questionnaires touching on similar issues, and the questionnaire was thoroughly pilot-tested before implementation to ensure face and content validity. Likewise, the questionnaire responses were nuanced and validated by the in-depth interviews. The interview citations were professionally language-edited but not back-translated.

## Conclusions

The Danish version of the POLST form is assessed by patients, families, physicians, and nurses as a useful model for obtaining and documenting Danish patients’ preferences for life-sustaining treatment. However, this needs to be confirmed in a larger-scale study.

## Supplementary Information

**Additional file 1.** A translation of the Danish POLST form.

**Additional file 2.** A translation of the patient evaluation questionnaire and physician, family, and nurse questionnaire, where they differ from the patient questionnaire.

## Data Availability

Data are available from the corresponding author on reasonable request.

## Abbreviations

ACP: Advance Care Planning
CPR: Cardiopulmonary Resuscitation
EOL: End-of-life
ICU: Intensive Care Unit
OPEN: Open Patient data Explorative Network
POLST (USA): Physician Orders for Life-Sustaining Treatment
POLST (DK): Patient Og Lægebeslutninger for den Sidste Tid. (English term "Patient And Physician decisions for End-of-Life")
QSR NVivo: A qualitative data analysis computer software produced by QSR
REDCap: Research Electronic Data Capture
ReSPECT: Recommended Summary Plan for Emergency Care and Treatment
