# Supplementary material for: Discussing patient preferences for levels of life-sustaining treatment: development and pilot testing of a Danish POLST form
Source: BMC Palliat Care. 2022 Jan 11;21:9. doi: 10.1186/s12904-021-00892-2 (PMC8749111; doi:10.1186/s12904-021-00892-2)
Supplement: Supplementary file 1 — Additional file 1. A translation of the Danish POLST form. [file 12904_2021_892_MOESM1_ESM.docx]

**The project document is NOT legally binding. Decisions must be documented in the patient´s medical record to be valid.**

| Patient-and-Physician Decisions for End-of-Life | | | | 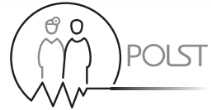 |
| --- | --- | --- | --- | --- |
| Patient last name: | | Patient first- and middle name: | Patient civil personal registration number: | |
| Address: | | Zip: / City: | Date form prepared: | |
| Family member participating in the conversation.  Name: Relationship to patient: | | | | |
| **A**  Check one | **Cardiopulmonary resuscitation** | | | |
|  | **☐ Attempt resuscitation.**  If it makes sense in terms of medical treatment.  **☐ Do not attempt resuscitation.**  Allow natural death. | | | |
| **B**  Check one | **Medical interventions** | | | |
|  | **☐ Comfort-focused treatment – primary goal is maximum comfort through symptom management.**  Relieve pain and suffering. Do not request transfer to hospital for life-prolonging treatment. If comfort needs cannot be met in current location, optionally transfer to hospital.  **☐ Selective treatment – primary goal is treating medical conditions with basic medical treatments.**  In addition to comfort-focused treatment use antibiotics, IV fluids, blood transfusions and support airways without use of mechanical ventilation. Transfer to hospital if needed. Generally avoid intensive care.  **☐ Full treatment – primary goal is life-prolonging treatment.**  In addition to comfort-focused treatment, use treatments as needed, transfer to hospital and optionally intensive care unit and mechanical ventilation if it makes sense in terms of medical treatment. | | | |
| **C**  Check one | **Nutrition** | | | |
|  | **☐If not capable of eating sufficiently for a longer period of time, administer feeding tube**  **☐If not capable of eating sufficiently for a longer period of time, do *not* administer feeding tube** | | | |
| **D** | **Documentation of conversation**  **Signature of patient**  These are my current wishes. I understand that completing this document is voluntary and that I can change my wishes at any time.  **Date: ________________ Signature: _____________________________________________**  **Signature of physician**  The patient has capacity. The above is consistent with the patient's current medical condition and wishes.  **Physician name** (capital letters): **Workplace:**  **­­____________________________________ _____________________________________**  **Date: _______________ Signature: ______________________________________________** | | | |

| **Additional comments and wishes** |
| --- |
|  |
| **Completing the project document: instructions for patients** |
| - Completing the project document is **always voluntary**. The document is relevant to you, if you have a serious illness, such as cancer, COPD or heart disease, to a severe degree, or if you are generally frail due to age. This is about you considering in advance what type of care you would like if you become unconscious or have a cardiac arrest. - The document is completed after a discussion between you (your family member), your general practitioner or hospital physician and nurse. You may have previously discussed the document with a nurse. The conversation includes your current diagnosis and treatment options if a medical emergency occurs. Depending on your current medical condition, there may be different treatments, such as mechanical ventilation, that will not be possible. - Together you reach a decision about what is important to you and the wishes you have for medical treatment and resuscitation. - The document records your – *current* – wishes for resuscitation and medical treatment*.* You can change your wishes at any time. |
| **Completing the project document: instructions for physicians and nurses** |
| - It is always voluntary for the patient to complete a project document. - The target group for POLST is patients with serious illnesses, such as cancer, COPD or heart disease, to a severe degree, or patients generally frail due to age. - A physician or a nurse can discuss the document with the patient. Both the patient and the physician must sign the document. |
| **Validity of decisions** |
| - The project document is NOT legally binding. The patient´s decisions, based on a discussion with the physician about preferences for medical treatment and resuscitation, must be documented in the patient´s medical record in accordance with the guidelines of the specific workplace. - The patient can at any time change his/her wishes. The changes are documented in the patient´s medical record. |
