# Supplementary material for: Discussing patient preferences for levels of life-sustaining treatment: development and pilot testing of a Danish POLST form
Source: BMC Palliat Care. 2022 Jan 11;21:9. doi: 10.1186/s12904-021-00892-2 (PMC8749111; doi:10.1186/s12904-021-00892-2)
Supplement: Supplementary file 2 — Additional file 2. A translation of the patient evaluation questionnaire and physician, family, and nurse questionnaire, where they differ from the patient questionnaire. [file 12904_2021_892_MOESM2_ESM.docx]

Patient

Questionnaire POLST* document

(*Patient and Physician Decisions for End-of-Life)

Date: _______________

**The first questions are about** personal information.

1. Age: ­­­­­­­­­__________ years
2. Gender: ☐ Male ☐ Female

**The next questions are about your** experience of the POLST document. Please read the questions and options for answers carefully and choose the answer that fits best.

1. Do you find the POLST document readable?

☐ Yes ☐ No

If no, why not?

1. Do you find the POLST document understandable?

☐ Yes ☐ No

If no, what is not understandable?

1. I found the level of information in the POLST document was:

☐ Much less than I needed

☐ A little less than I needed

☐ Appropriate

☐ A little more than I needed

☐ Much more than I needed

☐ Don´t know

1. Do you find there is information in the POLST document, that should either be added or removed?

☐ Yes ☐ No

If yes, what could be added and/or removed:

1. To which degree did you find that the POLST form was usable to talk about wishes for levels of life-sustaining treatment?
   1. The conversation with me (my family member) and the **physician**:

☐ To a very high degree

☐ To a high degree

☐ To some degree

☐ To a lesser degree

☐ Not at all

☐ Not applicable

Additional comments _________________________________________________

- 1. The conversation between me (my family member) and a **nurse**:

☐ To a very high degree

☐ To a high degree

☐ To some degree

☐ To a lesser degree

☐ Not at all

☐ Not applicable

Additional comments _________________________________________________

- 1. The conversation between me and my **family members**:

☐ To a very high degree

☐ To a high degree

☐ To some degree

☐ To a lesser degree

☐ Not at all

☐ Not applicable

Additional comments _________________________________________________

1. What do you find is good and/or less good about the POLST document?
2. Do you have other comments or ideas about the POLST document?

**Physicians, family members and nurses answered similar questionnaires, apart from the additional and/or adjusted questions added below:**

**Physician questionnaire:**

**The first questions are about** personal information.

1. Age: ­­­­­­­­­__________ years
2. Gender: ☐ Male ☐ Female
3. Profession:

☐ Hospital physician

☐ General practitioner

☐ Other

7: To which degree did you find that the POLST form useful in discussing patient´s wishes

for End-of-Life?

☐ To a very high degree

☐ To a high degree

☐ To some degree

☐ To a lesser degree

☐ Not at all

☐ Not applicable

Additional comments _________________________________________________

**Family member questionnaire:**

**The first questions are about** personal information.

1. Age: ­­­­­­­­­__________ years
2. Gender: ☐ Male ☐ Female
3. Relationship to patient:

☐ I am the patient´s spouse or partner

☐I am the patient´s son or daughter

☐I am the patient´s brother or sister

☐I am the patient´s father or mother

☐I am a relative of the patient not listed above

☐I am the patient´s friend

☐other _______________________________________

**Nursing staff questionnaire:**

**The first questions are about** personal information.

1. Age: ­­­­­­­­­__________ years
2. Gender: ☐ Male ☐ Female
3. Profession:

☐ Registered Nurse

☐ Nursing Assistants

☐ Social- and Health Care Helper

☐ Other
